# Effective lagrangian for axial anomaly and its applications in Dirac and Weyl semimetals

**DOI:** 10.1038/s41598-018-29676-0

**Published:** 2018-07-26

**Authors:** Chih-Yu Chen, C. D. Hu, Yeu-Chung Lin

**Affiliations:** Department of Physics, National Taiwan University, Taipei, Taiwan

## Abstract

A gauge invariant effective lagrangian for the fermion axial anomaly is constructed. The dynamical degree of freedom for fermion field is preserved. Using the anomaly lagrangian, the scattering cross section of pair production *γγ* → *e*^−^*e*^+^ in Dirac or Weyl semimetal is computed. The result is compared with the corresponding result from Dirac lagrangian. It is found that anomaly lagrangain and Dirac lagrangian exhibit the same **E** ⋅ **B** pattern, therefore the **E** ⋅ **B** signature may not serve a good indicator of the existence of axial anomaly. Because anomaly generates excessive right-handed electrons and positrons, pair production can give rise to spin current by applying gate voltage and charge current with depositing spin filters. These experiments are able to discern genuine anomaly phenomena.

## Introduction

Axial anomaly^1,2^, sometimes referred to as chiral anomaly in proper context, arises from the non-invariance of fermion measure under axial *γ*^5^ transformation^3^, it is a generic property of quantum fermion field theory, massive or massless. The realization of axial anomaly in condensed matter is first explored by Nielsen and Ninomiya^4^. It is argued that in the presence of external parallel electric and magnetic fields, there is net production of chiral charge and electrons move from left-handed (LH) Weyl cone to right-handed (RH) Weyl cone, the induced anomaly current causes magneto-conductivity prominent. Aji^5^ investigated pyrochlore iridates under magnetic field. He found that the energy dispersion is linear and the velocity is parallel to the applied magnetic field. External electric field which is also parallel to the magnetic field will introduce the imbalance between RH and LH particles and thus, Adler-Bell-Jackiw (ABJ) anomaly. Zyuzin *et al*.^6^ discussed time reversal and inversion symmetries of Weyl semimetal. They used multilayer systems as their model and calculated the current under external magnetic field. The origin of the nondissipative current was recognized as the ABJ anomaly. The experimental discoveries of Dirac and Weyl semimetals^7–9^ facilitate the opportunities for the observation. In recent experiments^10–13^ it is observed that the negative magnetoresistance is optimized when the applied electric field is parallel to the magnetic field, or the polarization vectors of the applied fields are perpendicular to each other. It is then argued that this is an evidence of anomaly as anomaly typically exhibits **E** ⋅ **B** behavior.

It is interesting to note that the A-phase superfluid of ^3^He possesses a similar type of chiral anomaly. As pointed out by Balatskii *et al*.^14^, the Fermi surface of ^3^He-A has two nodes at *ek*_*F*_**l** where **l** is the angular momentum of Cooper pairs and *e* = ±1 serving as the pseudo-charge of the fermions. It plays the role of chirality in ordinary fermion theory. The spins of Helium atoms are not relevant here. Therefore, instead of RH and LH states, there are states of opposite pseudo-charge near two nodes. Furthermore, there is a fictitious magnetic field ***B*** = ∇ × **l**. Balatskii *et al*.^14^ were able to show that the anomalous current is in the direction of the magnetic field by considering the *n* = 0 Landau levels. There are certain analogies between our theory and that in ref.^14^. For example, the function of *γ*^5^ is the same as the pseudo-charge *e* in the second term of their equation of motion (Eq. (2.9) in^14^). The axial current in this article is of the same characters as the chiral current of Nielsen and Ninomiya^4^ and the anomalous current of Balatskii *et al*.^14^.

Previous studies used Chern-Simons term^6^ to construct an effective lagrangian for axial anomaly in Weyl semimetals by integrating out the fermion degree of freedom, and thus treats the fermion field as a mean field of the background. The residual Chern-Simons term exhibits **E** ⋅ **B** form and is used to argue that it is a signature of axial anomaly in condensed matter. This approach leaves very little room for the study of electron transport properties as the degree of freedom for electron has been frozen out. It is the purpose of this work to construct an effective lagrangian which describes the axial anomaly in a fermion system, and keep the fermion field dynamical. It can be applied to high energy physics or condensed matter physics, such as Dirac semimetal or Weyl semimetal. With such an effective lagrangian which contains dynamical fermion field, it is easy to account for the effect of anomaly while keeping fermion dynamics explicit.

## Results

### Effective Lagrangian

Classical QED has both vector symmetry and axial symmetry, the QED lagrangian is invariant under *ψ* → *e*^−*iα*^*ψ* and $\psi \to {e}^{-i\beta {\gamma }^{5}}\psi$ respectively. If quantum effect is incorporated, the combination of vector symmetry and axial symmetry will be spoiled. Because vector symmetry is tied to particle number conservation, it must be strictly preserved, the axial symmetry is therefore chosen to be broken. The divergence of axial current $\bar{\psi }{\gamma }^{\mu }{\gamma }^{5}\psi$ is not equal to zero and is proportional to the amplitude of anomaly. The axial anomaly can be considered as a source generating axial current. It is noted that in the calculation of anomaly Feynman diagrams, what is concerned most is the nature of the external currents connected to the vertices, vector or axial, the actual material content, such as quarks of electrons, entering into the loop is not that essential to the result.

The construction of effective chiral anomaly lagrangian incorporating fermion follows direct analogy of the celebrated case *π*^0^ → *γγ*^15,16^. The relevant piece in the chiral anomaly lagragian for *π*^0^ → *γγ* takes the form of product of axial current and Chern-Simons term, which can be expressed by $(i/{f}_{\pi }){\partial }^{\mu }{\pi }^{0}{\varepsilon }_{\mu \nu \alpha \beta }{A}^{\nu }{\partial }^{\alpha }{A}^{\beta }$, where *f*_*π*_ = 93 *MeV* is the pion decay constant, $(i/{f}_{\pi }){\partial }^{\mu }{\pi }^{0}$ is the pion axial current and ${\varepsilon }_{\mu \nu \alpha \beta }{A}^{\nu }{\partial }^{\alpha }{A}^{\beta }$ is the Chern-Simons term for photon. The lagrangian piece obeys the demanded parity symmetry of QED. There are two vector fields, two partial derivatives, one pseudoscalar field and one Levi-Civita tensor, which is also a pseudoscalar, together they leave the lagrangian piece unchanged under parity operation. By replacing the pion axial current with the fermion axial current $\bar{\psi }{\gamma }^{\mu }{\gamma }^{5}\psi$, which is automatically parity symmetric, the chiral anomaly lagrangian for fermion is given by

1 $${L}_{\gamma \gamma }=g{e}^{2}{\varepsilon }_{\mu \nu \alpha \beta }\bar{\psi }{\gamma }^{\mu }{\gamma }^{5}\psi {A}^{\nu }{\partial }^{\alpha }{A}^{\beta },$$

where *e* is the electron charge, *g* is an effective coupling constant representing the quantum effect contributing to axial anomaly, *ψ* is fermion field, *A* is photon field and the subscript *γγ* means the vertex being a two-photon process. It is in form of axial-vector-vector (*AVV*) coupling in chiral anomaly. In principle, a generic *AVV* coupling could have various different forms. However, since photon is a vector current, the axial current can only arise from fermion. Moreover, the two photon fields and their associated momenta must be organized in form of Chern-Simons term so that the resulting form will reflect the topological nature of chiral anomaly.

From the dimensional analysis perspective, *L*_*γγ*_ is of dimension 4, *ψ* is of dimension 3/2, *A* and ∂ are of dimension 1, and hence *g* in *L*_*γγ*_ has to be of dimension −2. An effective theory with negative dimension coupling constant is only applicable to low energy regime. The application of this effective lagrangian to Dirac or Weyl semimetal conforms to this requirement as only physics of energy momentum around the fermi level is of interest in condensed matter.

The effective coupling constant *g* is the only parameter in such effective lagrangian. In principle it can be determined by fitting the scattering cross section or decay rate of a physical process described by the effective lagrangian. In meson physics, if a process arises from anomaly lagrangian, such as *π*^0^ → *γγ*, it will not receive contribution from the kinetic energy term as it is evident from parity argument. Strong and electromagnetic interactions respect parity symmetry, a meson is a pseudoscalar and therefore carries negative parity. Physical processes given rise from kinetic energy term must be present in even number of mesons in order to respect parity, while processes given rise from anomaly term typically have odd number of mesons as there is a Levi-Civita tensor which carries negative parity. Therefore anomaly process is free from background processes arising from kinetic energy term. This is not the case for fermion anomaly lagrangian in Eq. (2). Fermion typically manifest themselves in bilinear form of fermion fields in lagrangian, and the bilinear form of fermion current can either transform as vector current or axial current; the former appears in Dirac term and the later appears in anomaly term. As such, an electron-photon interaction could receive contributions from both Dirac term and anomaly term. Because of this mixing effect, using the scattering cross section or decay rate of a physical process to fit the effective coupling constant *g* is difficult as the contribution from the anomaly term is buried under the dominant Dirac term. Observation of axial anomaly needs to utilize other characteristics which only emerge in anomaly sector.

The term in Eq. (1) describes the coupling between two-photon and axial current. This term by itself is not gauge invariant. Following Witten trial and error approach^17^ for the construction of chiral anomaly lagrangian, a single-photon term has to be incorporated

2 $$\begin{array}{rcl}{L}_{A} & = & {L}_{\gamma \gamma }+{L}_{\gamma }\\  & = & g{e}^{2}{\varepsilon }_{\mu \nu \alpha \beta }\bar{\psi }{\gamma }^{\mu }{\gamma }^{5}\psi {A}^{\nu }{\partial }^{\alpha }{A}^{\beta }-ige{\varepsilon }_{\mu \nu \alpha \beta }\bar{\psi }{\gamma }^{\mu }{\gamma }^{5}{\partial }^{\nu }\psi {\partial }^{\alpha }{A}^{\beta }\mathrm{.}\end{array}$$

The lagrangian so constructed is gauge invariant. It is noted that the effective lagrangian retains the dynamical degree of freedom for fermions and hence the detailed behavior of individual fermion can be tracked and predicted. Together with the Dirac lagrangian, the kinetic energy term with electron-photon minimum coupling,

3 $${L}_{D}=i\bar{\psi }{\gamma }^{\mu }({\partial }_{\mu }+ie{A}_{\mu })\psi ,$$

they give an effective description of massless fermion interacting with photon incorporating the anomaly effect

4 $$L={L}_{D}+{L}_{A}.$$

It is noted that in *L*_*D*_ the fermion current coupled to photon field is a vector current, in contrast to the axial current in *L*_*A*_. The lagrangian is constructed on the premises of fermions being massless, however, it can also be applied to QED if a mass term is incorporated.

### Pair Production

The electron-positron pair production by two-photon scattering ($\gamma \gamma \to {e}^{-}{e}^{+}$) will be the focus of interest in this work. This process receives contributions from both Dirac and anomaly terms. This makes fermion anomaly phenomenon seemingly difficult to be observed as the genuine anomaly signal (theoretically suppressed by one-loop effect) will be overwhelmed by the corresponding Dirac term background process, which receives contributions from tree level diagrams. In particular, if physical observation measures the average collective behavior of such phenomenon, such as scattering cross section in which summing over final spin and average over initial spin have been performed, the anomaly effect might be completely lost.

Axial current inherits very different nature, in particular in chirality or helicity, compared with that of vector current. In Dirac or Weyl semimetal, the effective mass of electron is zero, and hence chirality, the eigenvalue of *γ*^5^ operator, and helicity, given by $\sigma \cdot {\bf{p}}/|p|$, are well defined and equal. Furthermore, due to fermion is massless in Dirac or Weyl semimetal, there is no mass term to mix RH fermion with LH fermion, processes arising from the Dirac term in Dirac lagrangian conserve helicity. The anomaly lagrangian serves as the sole source of and gives rise to excessive RH fermions. Utilizing this observation, it is possible to discern physical processes arising from Dirac term from those arising from anomaly term.

**Figure 1:**
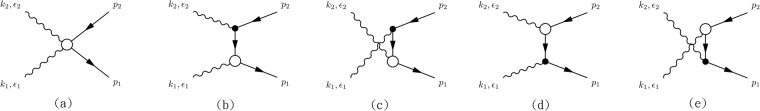
Feynman diagrams of *e*^−^*e*^+^ pair production by two-photon scattering involving anomaly lagrangian. (**a**) Arises from the two-photon contact term in Eq. (2), while the other diagrams are mixtures of one vertex from the single-photon term in *L*_*A*_ and one vertex from *L*_*D*_. The circle stands for the anomaly vertex and the solid dot stands for Dirac vertex.

The process of interest, $\gamma \gamma \to {e}^{-}{e}^{+}$, as shown in Fig. 1 bears certain resemblance to the cases studied in Nielsen and Ninomiya’s theoretical work^4^ and recent experiments on negative magnetoresistance^10–13^. It contains two photons, though not exactly the same as the constant applied fields in those cases, and it gives rise to *e*^−^*e*^+^ in axial current form; the scattering cross section might also depend upon the angle between the two photon polarization vectors. In the following calculation, the kinematic variables used in the system are *k*_1_ and *k*_2_ for the momenta of the incoming photons, *p*_1_ and *p*_2_ for the momentum of the outgoing electron and positron, respectively. *ε*_1_ and *ε*_2_ are the polarization vectors for photons. The electron and positron spinors are denoted by *u*(*p*_1_, *s*_1_) and *v*(*p*_2_, *s*_2_), respectively. Due to all the particles in the system are massless, the kinematics relations are very simple, ${k}_{1}^{2}={k}_{2}^{2}={p}_{1}^{2}={p}_{2}^{2}=0$. The polarization vectors are transverse, and the temporal gauge, *A*_0_ = 0, is adopted, therefore in the CoM frame (${k}_{1}^{j}=-{k}_{2}^{j}$) they lead to ${k}_{1}\cdot {\varepsilon }_{1}={k}_{2}\cdot {\varepsilon }_{2}=0$ and ${k}_{1}\cdot {\varepsilon }_{2}={k}_{2}\cdot {\varepsilon }_{1}=0$. The condition of being transverse for polarization is not equivalent to Lorenz gauge, nor Coulomb gauge where the gauge condition reads ${k}_{\mu }{A}^{\mu }=0$ and $\nabla \cdot {\bf{A}}=0$, in which *A*_*μ*_ = *A*_*μ*_(*x*). Also because the fermion fields are massless, the normalization conditions obey ${\sum }_{s}u(p,s)\bar{u}(p,s)={\sum }_{s}v(p,s)\bar{v}(p,s)=\rlap{/}{p}$ and the propagator becomes $({\rlap{/}{p}}_{1}-{\rlap{/}{k}}_{1})$ /(2*p*_1_ ⋅ *k*_1_).

The amplitudes for the Feynman diagrams depicted in Fig. 1 are given by

5 $$\begin{array}{rcl}{M}_{1} & = & g{e}^{2}\bar{u}({p}_{1},{s}_{1}){\gamma }^{\mu }{\gamma }^{5}v({p}_{2},{s}_{2}){\varepsilon }_{\mu \nu \alpha \beta }({\varepsilon }_{1}^{\nu }{k}_{2}^{\alpha }{\varepsilon }_{2}^{\beta }+{\varepsilon }_{2}^{\nu }{k}_{1}^{\alpha }{\varepsilon }_{1}^{\beta }),\\ {M}_{2} & = & g{e}^{2}\bar{u}({p}_{1},{s}_{1}){\gamma }^{\mu }{\gamma }^{5}{\varepsilon }_{\mu \nu \alpha \beta }{p}_{1}^{\nu }{k}_{1}^{\alpha }{\varepsilon }_{1}^{\beta }\frac{{\rlap{/}{p}}_{1}-{\rlap{/}{k}}_{1}}{2{p}_{1}\cdot {k}_{1}}{\rlap{/}{\varepsilon }}_{2}v({p}_{2},{s}_{2}),\\ {M}_{3} & = & g{e}^{2}\bar{u}({p}_{1},{s}_{1}){\gamma }^{\mu }{\gamma }^{5}{\varepsilon }_{\mu \nu \alpha \beta }{p}_{1}^{\nu }{k}_{2}^{\alpha }{\varepsilon }_{2}^{\beta }\frac{{\rlap{/}{p}}_{1}-{\rlap{/}{k}}_{2}}{2{p}_{1}\cdot {k}_{2}}{\rlap{/}{\varepsilon }}_{1}v({p}_{2},{s}_{2}),\\ {M}_{4} & = & g{e}^{2}\bar{u}({p}_{1},{s}_{1}){\rlap{/}{\varepsilon }}_{1}\frac{{\rlap{/}{p}}_{1}-{\rlap{/}{k}}_{1}}{2{p}_{1}\cdot {k}_{1}}{\gamma }^{\mu }{\gamma }^{5}{\varepsilon }_{\mu \nu \alpha \beta }{p}_{2}^{\nu }{k}_{2}^{\alpha }{\varepsilon }_{2}^{\beta }v({p}_{2},{s}_{2}),\\ {M}_{5} & = & g{e}^{2}\bar{u}({p}_{1},{s}_{1}){\rlap{/}{\varepsilon }}_{2}\frac{{\rlap{/}{p}}_{1}-{\rlap{/}{k}}_{2}}{2{p}_{1}\cdot {k}_{2}}{\gamma }^{\mu }{\gamma }^{5}{\varepsilon }_{\mu \nu \alpha \beta }{p}_{2}^{\nu }{k}_{1}^{\alpha }{\varepsilon }_{1}^{\beta }v({p}_{2},{s}_{2}\mathrm{).}\end{array}$$

Summing over all the final spin, the transition matrix is given by

6 $$\sum _{{s}_{1},{s}_{2}}|M{|}_{A}^{2}=32{g}^{2}{e}^{4}{k}^{4}{\sin }^{2}\varphi ,$$

where *k* is the photon energy in the CoM frame, *ϕ* is the angle between the polarization vectors of two incoming photons and the subscript A denotes the contribution arising from anomaly. The corresponding differential cross section is then

7 $${(\frac{d\sigma }{d{\rm{\Omega }}})}_{A}=2{\alpha }^{2}{g}^{2}{k}^{4}{\sin }^{2}\varphi ,$$

where *α* is the fine structure constant.

It is interesting to note that the contributions from ${M}_{2}\sim {M}_{5}$ terms (see Fig. 1), contributions arising from the mixture of a single-photon vertex from anomaly and a single-photon vertex from Dirac term, all vanish in the final transition matrix element. The transition matrix receives contribution only from *M*_1_, Feynman diagram (a) in Fig. 1, which arises from the two-photon contact term in the *L*_*A*_. It is a reminiscence of the *γγ* → *π*^0^*π*^+^*π*^−^ process in pion physics. It receives contribution only from the two-photon contact term in chiral anomaly. All the contributions arising from single-photon vertex *γ* → *π*^0^*π*^+^*π*^−^ which exhibits in the same chiral anomaly lagrangian, together with another photon absorbed by either *π*^−^ or *π*^+^ in the aforementioned vertex cancel each other because the amplitudes are the same but the signs of electromagnetic coupling are opposite.

The transition matrix depends only on the photon energy *k* and the angle *ϕ* between two polarization vectors. It is isotropic in all directions, no angular dependence on the angles between the momenta of photons and the electron, unlike what is observed in the negative magnetoresistance experiments in which the axial current tends to move along the direction of aligned **E** and **B**. This is easy to be understood as the two photons convert to *e*^−^*e*^+^ pair axial current after the scattering process, they will no longer affect the direction of axial current. But the transition matrix is optimized when $\varphi =\pi \mathrm{/2}$, which means that the anomaly process prefers to have polarization vectors being perpendicular to each other, or **E** is parallel to **B**. This result seems to bolster previous theoretical and experimental arguments that **E** ⋅ **B** is the signature of anomaly.

**Figure 2:**
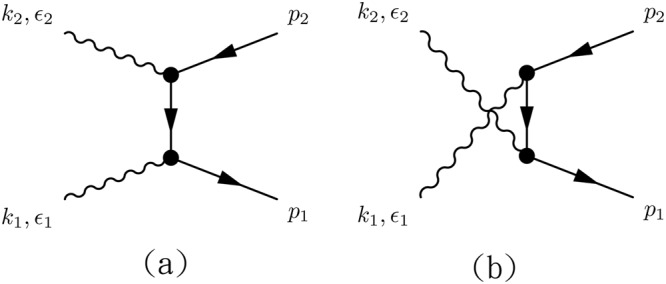
The Feynman diagrams for pure Dirac term contributions.

As aforementioned, the Dirac term also contributes to the *γγ* → *e*^−^*e*^+^ process. There are two Feynman diagrams (see Fig. 2) which contributes to the process. Their amplitudes read

8 $$\begin{array}{rcl}{M}_{1}^{D} & = & {e}^{2}\bar{u}({p}_{1},{s}_{1}){\rlap{/}{\varepsilon }}_{1}\frac{{\rlap{/}{p}}_{1}-{\rlap{/}{k}}_{1}}{2{p}_{1}\cdot {k}_{1}}{\rlap{/}{\varepsilon }}_{2}v({p}_{2},{s}_{2}),\\ {M}_{2}^{D} & = & {e}^{2}\bar{u}({p}_{1},{s}_{1}){\rlap{/}{\varepsilon }}_{2}\frac{{\rlap{/}{p}}_{1}-{\rlap{/}{k}}_{2}}{2{p}_{1}\cdot {k}_{2}}{\rlap{/}{\varepsilon }}_{1}v({p}_{2},{s}_{2}),\end{array}$$

where superscript *D* represents Dirac. The transition matrix element then can be simply derived as before

9 $$|M{|}_{D}^{2}=\frac{{e}^{4}}{8{\pi }^{2}}{\sin }^{2}\varphi .$$

The corresponding differential cross section is given by

10 $${(\frac{d\sigma }{d{\rm{\Omega }}})}_{D}=\frac{{\alpha }^{2}{\sin }^{2}\varphi }{2{k}^{2}}\mathrm{.}$$

Contribution arising from the Dirac term exhibits the same angular dependence as that from the anomaly term. Moreover, as anomaly term is of one-loop order effect, as a rule of thumb, it is typically one order of magnitude smaller than the tree level contribution. The signal of anomaly is overwhelmed by the Dirac contribution for *γγ* → *e*^−^*e*^+^. The reason why this is happening is because originally anomaly is of axial current and Dirac term is of vector current in nature, which are discernible in this regard. But when summing over all the final spins of fermion in the calculation, the pattern which originally could be used to tell the difference is averaged out. However, the genuine anomaly effect still can be detected. The details are presented in next subsection.

### Proposed Experiments

**Figure 3:**
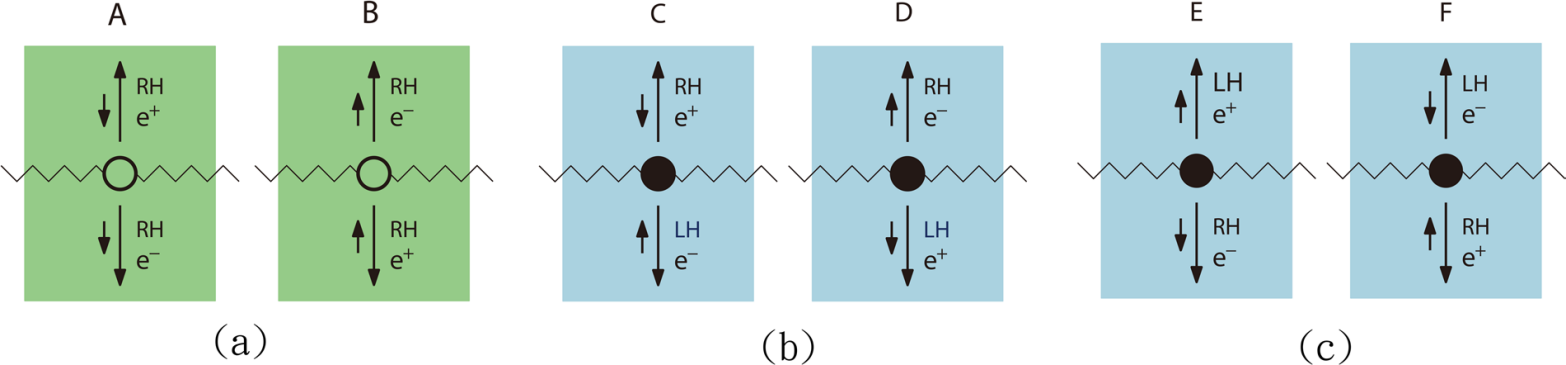
(**a**) The events of anomaly. The chirality is not conserved. (**b** and **c**) The events due to the Dirac term. The chirality is conserved. *e*^+^ and *e*^−^ denote positrons (holes) and electrons respectively. RH and LH denote right-handed and left-handed particles. Long arrows indicate the directions of momenta of fermions and wiggle lines denote photons. Short arrows indicate the directions of spins.

Chirality is conserved in the Dirac term but not in the anomaly term. As a result, net RH electron and RH positron are only created in the axial current generated by anomaly. This is illustrated in Fig. 3(a) where two equally likely events A and B are shown. On the other hand, there are four similar events which are generated by the Dirac term. They are events C, D, E and F in Fig. 3(b and c). To interpret them more clearly, the spin directions of all the particles are explicitly depicted so that one can tell the helicities of various particles. For the massless fermions in our case, the chirality and helicity are the same. A positron can also be viewed as a hole. Similarly, its momentum, spin and chirality are opposite to those of the missing electron. Note also that for antifermions, the helicity and chirality are defined oppositely to those of fermions, namely, the eigenvalue of $\sigma \cdot {\bf{p}}/|p|$ is −1 in the RH state of antifermions. A RH positron (hole) can be understood as a filled LH electron band with one electron removed. For example, in the event A in Fig. 3(a), the RH positron is equivalent to a filled band with one spin-up, negative-momentum electron missing. It is easily seen that the charge current produced in event A is canceled by that in event B. Since the transition matrix elements are isotropic, the charge current produced by electrons cancels that given rise by positrons in average and it seems no significant result can be extracted.

**Figure 4:**
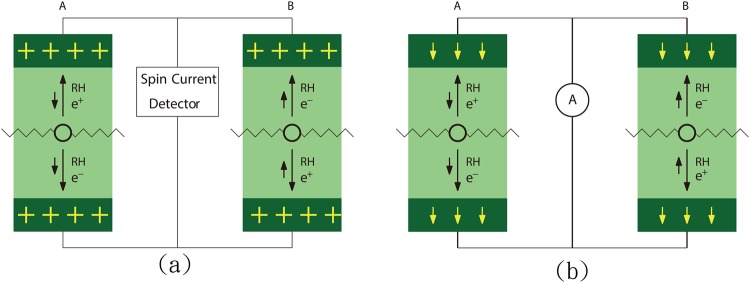
Proposed experimental setups for observing the effect of anomaly. (**a**) The dark areas indicate the places where positive gate voltage is applied. (**b**) The dark areas indicate the places where spin-down spin filters are deposited.

Experiments which are able to detect the axial current, or excessive RH electrons and positrons, generated by anomaly are then desired and proposed. Positive gate voltage is applied at both the upper end and the lower end (dark regions) as shown in Fig. 4(a). Negatively charged electrode can be placed under the sample so as to collect positrons. The goal of this is to suppress the upward positron current in event A and downward positron current in event B. There is still no net charge current, but there is net spin current. On the other hand, four similar events generated by the Dirac term, C, D, E and F in Fig. 3(b and c) have equal probability, and hence the resulting current vanishes due to complete cancellation even if the gate voltage mentioned above is applied. One can conclude that if spin current is observed in Fig. 4(a), it is definitely caused by axial anomaly. In fact, if the circuit is removed, there will be accumulation of up-spins at the upper end and down-spins at the lower end. They can be detected by magneto-optic Kerr effect.

In another setup, shown in Fig. 4(b), spin filters^18^ are deposited on upper and lower side (dark regions) of the sample, both with downward spins. This will reduce the resistance of the current in event A and enhance the resistance of the current in event B. Thus the contribution of event B is suppressed and charge current can be detected. Conventional spin filters can produce current with spin polarization as high as 50%^19^. More recent experiments showed that it can be nearly 100%. For a review, see for example, the work of Moodera, Santos and Nagahama^20^. Hence, it can give rise to significant spin-polarized charge current. Since the contributions from the Dirac term completely cancel each other, any measured signal comes from the axial anomaly. Even partially polarized current suffices the proof of the existence of axial current easily.

## Conclusion

In summary, a gauge invariant effective lagrangian for axial anomaly is constructed. It retains the dynamical degree of freedom of electron field and hence detailed electron behavior can be calculated and predicted. Using such effective anomaly lagrangian, it is found that the scattering transition matrix of *γγ* → *e*^−^*e*^+^ arising from the Dirac term and the anomaly lagrangian behave the same. As a result, the often-mentioned **E** ⋅ **B** signature may not serve as an evidence of axial anomaly in Dirac or Weyl semimetal. Experiments which can discern the physical effects of vector current generated by Dirac term and axial current generated by anomaly are proposed. It is interesting that in the presence of axial current, by applying electric gate voltage, it gives rise to spin current; and by applying magnets, it produces charge current.

## References

[1] Adler SL (1969). Axial-vector vertex in spinor electrodynamics. Phys. Rev..

[2] Bell JS, Jackiw R (1979). A pcac puzzle:*π*^0^ → *γγ* in the σ-model. Il Nuovo Cimento A.

[3] Fujikawa K (1979). Path-integral measure for gauge-invariant fermion theories. Phys. Rev. Lett..

[4] Nielsen HB, Ninomiya M (1983). The Adler-Bell-Jackiw anomaly and Weyl fermions in a crystal. Phys. Lett. B.

[5] Aji V (2012). Adler-Bell-Jackiw anomaly in Weyl semimetals: Application to pyrochlore iridates. Phys. Rev. B.

[6] Zyuzin AA, Wu S, Burkov AA (2012). Weyl semimetal with broken time reversal and inversion symmetries. Phys. Rev. B.

[7] Liu ZK (2014). Discovery of a three-dimensional topological Dirac semimetal, Na_3_Bi. Science.

[8] Liu ZK (2014). A stable three-dimensional topological Dirac semimetal Cd_3_As_2_. Nature Materials.

[9] Lv BQ (2015). Experimental discovery of Weyl semimetal TaAs. Phys. Rev. X.

[10] Huang X (2015). Observation of the chiral-anomaly-induced negative magnetoresistance in 3d weyl semimetal TaAs. Phys. Rev. X.

[11] Xiong J (2015). Evidence for the chiral anomaly in the dirac semimetal Na_3_Bi. Science.

[12] Li C-Z (2015). Giant negative magnetoresistance induced by the chiral anomaly in individual Cd_3_As_2_ nanowires. Nature Communications.

[13] Li H (2016). Negative magnetoresistance in Dirac semimetal Cd_3_As_2_. Nature Communications.

[14] Balatskii A, Volovik G, Konyshev V (1986). On the chiral anomaly in superfluid ^3^He-A. JETP.

[15] Carroll SM, Field GB, Jackiw R (1990). Limits on a lorentz- and parity-violating modification of electrodynamics. Phys. Rev. D.

[16] Lin Y-C (1995). Prediction of the anomalous magnetic moment of the nucleon from the nucleon anomaly. Phys. Lett. B.

[17] Witten E (1983). Global aspects of current algebra. Nucl. Phys. B.

[18] Filipe A (1998). Spin-dependent transmission of electrons through the ferromagnetic metal base of a hot-electron transistorlike system. Phys. Rev. Lett..

[19] Tedrow PM, Meservey R (1973). Spin polarization of electrons tunneling from films of Fe, Co, Ni, and Gd. Phys. Rev. B.

[20] Moodera JS, Santos TS, Nagahama T (2007). The phenomena of spin-filter tunnelling. Journal of Physics: Condensed Matter.

